# A Photocontrolled Molecular Rotor Based on Azobenzene-Strapped Mixed (Phthalocyaninato)(Porphyrinato) Rare Earth Triple-Decker

**DOI:** 10.3390/molecules30020326

**Published:** 2025-01-15

**Authors:** Wenxin Lu, Tiantian Mu, Yuehong Zhang, Bo Chen, Huantao Guo, Luyang Zhao, Peng Wang, Yongzhong Bian

**Affiliations:** 1 College of Chemical and Biological Engineering, Shandong University of Science and Technology, Qingdao 266590, China; lwx08lwx@126.com (W.L.); mtt010109@163.com (T.M.); cb2000706@163.com (B.C.); ght200027@163.com (H.G.); 2 Beijing Key Laboratory for Science and Application of Functional Molecular and Crystalline Materials, Department of Chemistry and Chemical Engineering, School of Chemistry and Biological Engineering, University of Science and Technology Beijing, Beijing 100083, China; 3 School of Advanced Manufacturing, Guangdong University of Technology, Jieyang 522000, China; yuehong@gdut.edu.cn; 4 State Key Laboratory of Biochemical Engineering, Institute of Process Engineering, Chinese Academy of Sciences, Beijing 100190, China; qczgzly@126.com

**Keywords:** molecular rotor, porphyrin, phthalocyanine, azobenzene, rare earth

## Abstract

Effectively regulating the rotary motions of molecular rotors through external stimuli poses a tremendous challenge. Herein, a new type of molecular rotor based on azobenzene-strapped mixed (phthalocyaninato)(porphyrinato) rare earth triple-decker complex **Azo-1** is reported. Electronic absorption and ^1^H NMR spectra manifested the reversible isomerization of the rotor **Azo-1** between the *trans* configuration and the *cis* configuration. The rotational behavior of phthalocyanine rotator in two configurations were investigated by VT-^1^H NMR experiments, and the results indicated that the phthalocyanine rotator possessed a smaller rotational energy barrier in the *cis* isomer than in the *trans* isomer, which was also supported by DFT calculations. This result demonstrates that the rotation of phthalocyanine rotator in (phthalocyaninato)(porphyrinato) rare earth triple-decker complex can be successfully modulated by photo-isomerization via altering irradiation.

## 1. Introduction

Natural biomolecular machines, such as kinesin motor proteins, ATP synthase and cellular flagella, sustain essential life processes through their operation [1]. Inspired by these biomolecular machines, the design and construction of artificial molecular machines have attracted worldwide interest [2]. From this perspective, the efforts made in the synthesis of molecular shuttles, molecular rotors, molecular elevators, and molecular motors have brought new prospects to the field of synthetic molecular machines [3,4,5]. Among the diverse reports on molecular machines, particular attention has been focused on the rotational motions, since the effective control of the rotational molecular motion can improve the functional performances and expand the applications [6,7,8,9].

Molecular rotors, a class of molecular machines composed of the rotator and the stator, which undergo relative rotation, not only exhibit applications in material chemistry [10,11] and nanotechnology [12,13], but also have extensive utilization in fields such as membrane engineering [14,15] and biological detection [16,17,18]. Various kinds of fascinating molecular rotors have been reported, such as molecular rotors embedded in MOFs [19,20] or COFs [21], gyroscop-like molecular rotors [22,23], turnstile-like molecular rotors [24] and molecular rotors in nanosheets [25,26]. Nonetheless, for the development of applications of molecular rotors in fluorescence sensing [27,28,29,30], CO_2_ sorption [31], molecular probes [32], and disease diagnosis [33,34], efficacious control over their rotary motions remains rather crucial. Consequently, the key challenge in the proximate future is thereby to regulate their rotation by means of external stimuli and to establish novel functions.

Azobenzene, which possesses two distinct isomeric configurations, namely *cis* and *trans* isomer, undergoes a reversible transformation between the *trans* and the *cis* isomer upon irradiation, with the *trans* isomer displaying an elongated configuration while the *cis* isomer presents a compact one [35,36,37]. Azobenzene is prevalently used as the photoconversion unit mainly due to its characteristics, such as rapid photo-isomerization, high reversibility, low photobleaching, and facile synthesis [38,39,40]. These advantages render azobenzene derivatives as having extensive applications, ranging from materials science to biology [41,42,43,44].

Multi-decker porphyrin-phthalocyanine complexes have captured considerable interest among researchers, not only for their optical and electrochemical properties [45,46,47], but also for their complete rotor/oscillator system for constructing single-molecule rotors [48,49]. Herein, we report a new type of molecular rotor based on a mixed (phthalocyaninato)(porphyrinato) rare earth triple-decker complex, with an azobenzene-linked bisporphyrin as the stator and a phthalocyanine as the rotator. The rotational barrier can be adjusted by irradiation-induced reversible isomerization.

## 2. Results

### 2.1. Molecular Design and Synthesis

The (phthalocyaninato)(porphyrinato) rare earth triple-decker complexes are prototypical molecular rotors, in which the outer phthalocyanine or porphyrin ligand acts as the stator and the inner ligand functions as the rotator. Furthermore, the triple-deckers possess excellent thermal stability [47], which is conducive to the modulation of molecular rotors by external stimuli. Azobenzene constitutes one of the most prevalent light-responsive molecular switches and it can be transformed between two isomers (*cis* and *trans*) with different structures [35]. The *trans* isomer assumes an expansive configuration, whereas the *cis* isomer takes on a compact configuration [36], and this feature makes it widely used in the construction of molecular machines. In this work, azobenzene was selected to attach the triple-decker unit and adjust the rotational behavior of the phthalocyanine rotator.

**Scheme 1:**
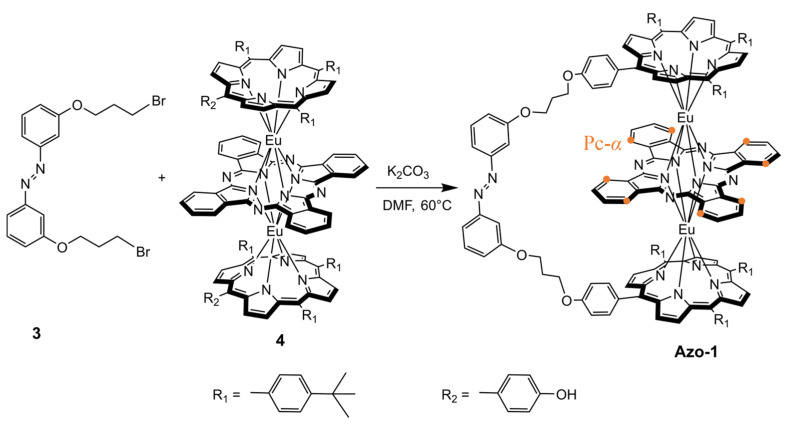
Synthesis of mixed (phthalocyaninato)(porphyrinato) rare earth triple-decker complex **Azo-1** and the orange small dots represent the positions of the *α* hydrogen atoms of phthalocyanine.

**Scheme 2:**
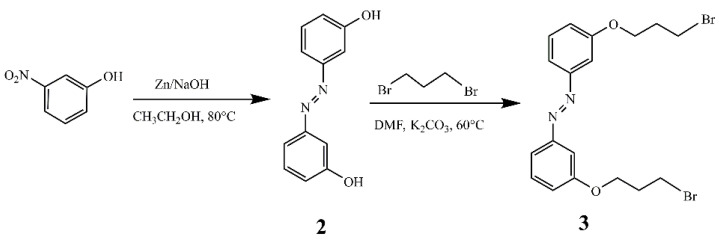
Synthesis of 4,4’-dihydroxylazobenzene and 4,4ʹ-di(3-bromopropoxy) azobenzene.

**Scheme 3:**
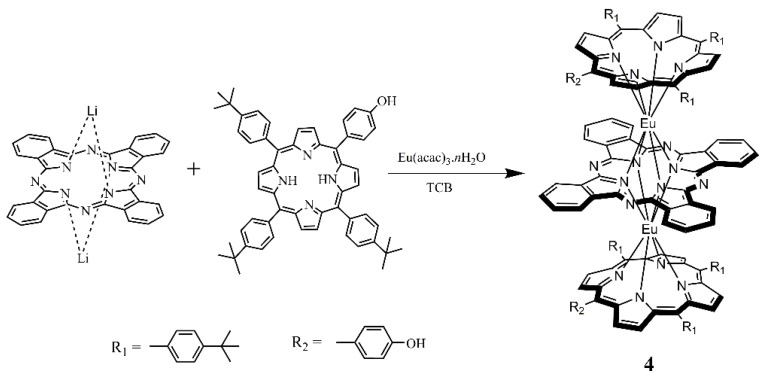
Synthesis of (TTBPP)Eu(Pc)Eu(TTBPP) (**4**).

The azobenzene-strapped mixed (phthalocyaninato)(porphyrinato) rare earth triple-decker complex **Azo-1** was synthesized by a Williamson’s coupling reaction of 4,4′-di(3-bromopropoxy) azobenzene (**3**) with (TTBPP)Eu(Pc)Eu(TTBPP) (**4**) in 21% yield (Scheme 1) and 4,4′-di(3-bromopropoxy) azobenzene was obtained by means of a similar reaction (Scheme 2). (TTBPP)Eu(Pc)Eu(TTBPP) was obtained by a one pot method with Eu(TTBPP)(acac) and Li_2_Pc in 14% yield (Scheme 3). **Azo-1** and its intermediates were adequately characterized by MS, NMR, and electronic absorption spectra (see the Supplementary Information for details, Figures S1–S6). The *cis*–*trans* reversible isomerization of **Azo-1** was investigated by electronic absorption spectroscopy and NMR experiments.

### 2.2. Electronic Absorption Spectroscopy

**Figure 1:**
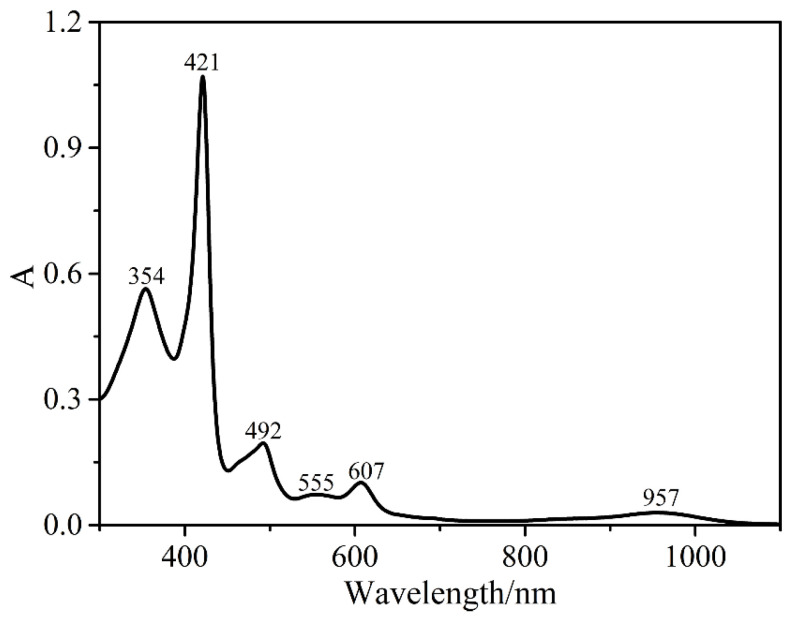
Electronic absorption spectra of **Azo-1** measured at 298 K (5.0 × 10^−6^ M in toluene).

The electronic absorption spectrum of compound **Azo-1** was measured in toluene and in the range of 300–1100 nm, which shows the typical spectrum of mixed (phthalocyaninato) (porphyrinato) rare earth triple-decker complexes. As can be seen in Figure 1, the absorption at 354 and 421 nm are the Soret bands of phthalocyanine and porphyrin, respectively. The absorptions at 492, 555, 607, and 957 nm are the Q bands including the characteristics of porphyrin and phthalocyanine. In the triple-decker complex, the close proximity of the three conjugated π-systems in a face-to-face configuration induces a splitting of the monomer molecular orbitals. The near IR absorption band at 957 nm can be attributed to the electronic transition from the first anti-bonding highest occupied molecular orbital (HOMO) to the first bonding lowest unoccupied molecular orbital (LUMO), and the absorption band at 607 nm can be attributed to the transition from the second non-bonding HOMO to the second non-bonding LOMO. The Q bands at 492 and 555 nm are derived from the electronic transition involving molecular orbitals with mixed porphyrin and phthalocyanine [50,51]. The two characteristic absorption bands of azobenzene are at about 320 and 450 nm [35] and were overlapped with the Soret bands of porphyrin and phthalocyanine.

### 2.3. The Cis–Trans Reversible Isomerization

**Figure 2:**
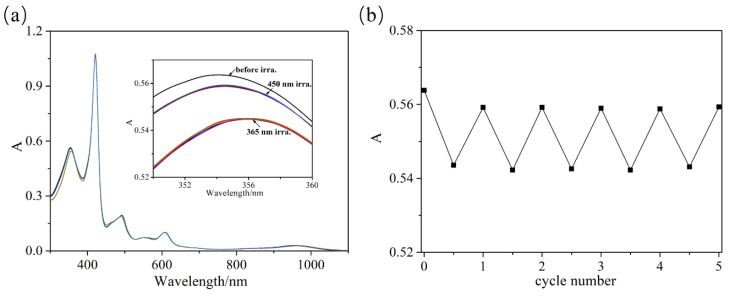
Electronic absorption spectra (**a**) and absorption changes at λ = 354 nm (**b**) of **Azo-1** (5.0 × 10^−6^ M in toluene) under light irradiation at λ = 365 nm or λ = 450 nm. Inset: expanded version of part of the spectra.

Azobenzene derivatives exhibit reversible photoconversion capability, which is ascribed to the isomerization of *cis* and *trans* configuration when exposed to ultraviolet and visible light alternately. The photochemical isomerization process of **Azo-1** was monitored by electronic absorption spectroscopy, with a wavelength range from 300 to 1100, nm at room temperature. As can be seen in Figure 2 and Figure S7, upon the continuous irradiation of **Azo-1** with 365 nm light for 180 s, the absorbance at 354 nm decreased, accompanied by an increase in the absorption at 451 nm, and no further changes were induced with extended UV light exposure. Subsequent irradiation with 450 nm light for 180 s resulted in an increase in the absorbance of **Azo-1** at 354 nm and a decrease at 451 nm; it then reverted to its original state. The absorption band at 354 nm corresponds to the π-π* transition of the stable *trans* isomers and the peak at 451 nm is due to the n-π* transition of the unstable *cis* isomers of azobenzene derivatives [36]. In addition, the absorption band under bathochromic shifted from 354 to 356 nm when irradiated with 365 nm light and reverted back to the original position upon 450 nm light irradiation. These spectral alterations provide unequivocal evidence for the efficient transformation from *trans* to *cis* photoisomerization upon irradiation with 365 nm light and the subsequent back isomerization from *cis* to *trans* isomer after irradiation with 450 nm light. **Azo-1** was irradiated alternately with 365 and 450 nm light for five cycles, and the unaltered absorption band revealed no indication of photodegradation, which signified that the *trans*–*cis* photoisomerization was stable and reversible. When irradiated with 365 nm light, the absorbance of (TTBPP)Eu(Pc)Eu(TTBPP)(compound **4**) scarcely changed (Figure S8), thereby demonstrating that compound **4** is photostable.

**Figure 3:**
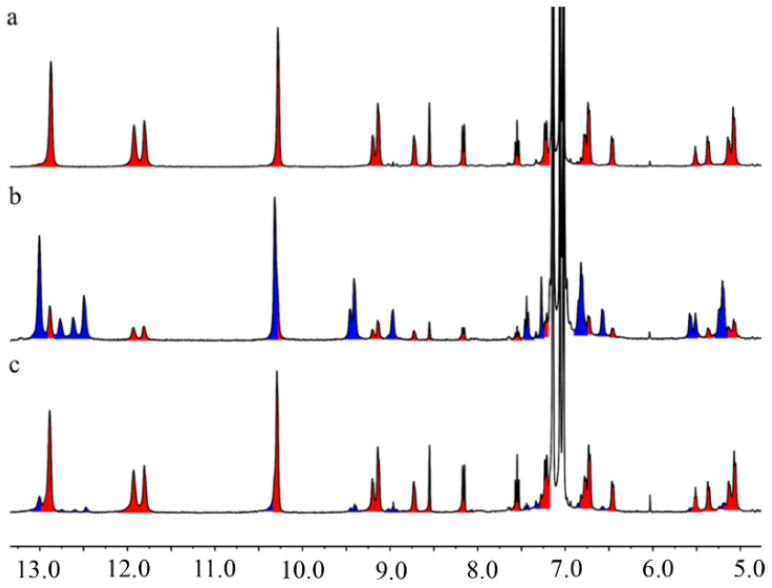
^1^H NMR spectra of **Azo-1** ([D_8_] toluene, 298 K): (**a**) before irradiation, (**b**) after irradiation with 365 nm light for 20 min, (**c**) after further irradiation with 450 nm light for 20 min. The red region represents the characteristic signals of the *trans* isomer and the blue region represents the characteristic signals of the *cis* isomer.

The spectrum presented in Figure 3 depicts the ^1^H NMR spectrum of **Azo-1** for further investigation of the *trans*–*cis*–*trans* photoisomerization process. Before irradiation, the ^1^H NMR signals at 12.87 and 10.28 ppm correspond to the *α* and *β* protons of the phthalocyanine ligand, respectively, whilst other signals within the range of 12.00 to 9.00 ppm signify the aromatic protons on the substituted benzene rings of porphyrin (Figure 3a). This suggests that **Azo-1** exists solely in the *trans* conformation in solution at the outset. After irradiation with 365 nm light for 20 min, the resultant ^1^H NMR spectrum, as depicted in Figure 3b, reveals attenuated characteristic peaks of the *trans* isomer, concomitant with the emergence of new signal peaks at lower field positions. Specifically, in the lower field positions, rather than 12.87 and 10.28 ppm, new signal peaks emerge at 13.00 and 10.32 ppm, respectively, which are the characteristic peaks of the *cis* isomer. The isomerization from the *trans* configuration to the *cis* configuration brings the two porphyrins on the azobenzene moiety into close proximity [52] and gives rise to a weakened shielding effect and causes the *α* and *β* protons of the phthalocyanine, as well as the aromatic protons on the substituted benzene ring of porphyrin, to exhibit slight downfield shifts. Through comparing the integral areas of the signals at 13.00 and 12.87 ppm in Figure 3b, the ratio of *cis*/*trans* isomers is estimated to be 76:24 and it remains unaltered even upon prolonged irradiation. As reported, the thermodynamically metastable *cis*-azobenzene has a half-life of 2 days for unmodified azobenzene [43]. For **Azo-1**, due to the fact that the two benzene rings in azobenzene are restricted by the alkyl chain, the thermal *cis* to *trans* isomerization demands a greater amount of heat, and thus the *cis* isomer remains stable under ambient temperature and darkness. The sample was subsequently irradiated with 450 nm light for 20 min and the consequent ^1^H NMR spectrum is depicted in Figure 3c. Notably, the signals associated with the *cis* isomer have basically vanished, while the signals attributed to the *trans* isomer prevail and the ratio of *trans*/*cis* isomers is up to 92:8. These spectral changes provide further evidence for the reversible *trans*–*cis* photoisomerization of **Azo-1**.

### 2.4. Investigating the Rotational Dynamics of Photo-Responsive Rotor Through Variable Temperature ^1^H NMR Spectroscopy

**Figure 4:**
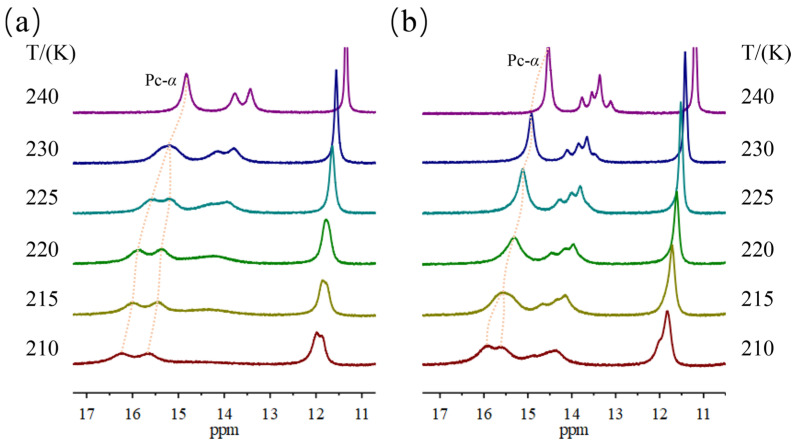
^1^H NMR spectra of **Azo-1** ([D_8_] toluene) in the range of 210–240 K: (**a**) *trans* configuration and (**b**) *cis* configuration.

The rotational behavior of the rotor in the *trans* state was initially investigated in the temperature range of 205–298 K in [D_8_] toluene, as shown in Figure 4a and Figure S9. By scrutinizing the *α* proton signals of phthalocyanine and observing their temperature-dependent fluctuations, the rotational dynamics displayed by the phthalocyanine rotator have been explored. At 298 K, the NMR spectrum of *trans*-**Azo-1** reveals a sharp peak at 12.91 ppm, representing the *α* protons of phthalocyanine. This suggests that the rotational exchange among the eight nonequivalent *α* protons of phthalocyanine is occurring at a rate faster than the NMR time scale, indicating the swift rotation of the phthalocyanine rotator. As the temperature decreases, the sharp singlet of the *α* protons of phthalocyanine gradually broadens and shifts towards the low field. When the temperature drops to 225 K, it splits into two peaks with equal areas, located at chemical shifts of 15.59 and 15.19 ppm, respectively. This indicates that, at such low temperature, the rotation rate of the phthalocyanine rotator is sufficiently slow to be detected on the ^1^H NMR spectroscopic time scale. The gradual transition from swiftness to slowness within this dynamic process enabled us to attain the coalescence temperature (*T_c_*-*trans*) of 230 K for *trans*-**Azo-1**. Similarly, the rotational behavior of the rotor in the *cis* configuration was also investigated as a function of temperature using VT-^1^H NMR spectroscopy (Figure 4b and Figure S10). As the temperature decreases, the sharp signal of the phthalocyanine *α* protons gradually broadens and shifts to a low field. When the temperature decreases to 210 K, it also splits into two peaks with equal integral areas. Consequently, the coalescence temperature (*T_c_*-*cis*) of 215 K was obtained for *cis*-**Azo-1**. The coalescence temperature of *cis*-**Azo-1** is lower than that of *trans*-**Azo-1**; therefore, it can be inferred that the rotation of phthalocyanine is easier in the *cis* isomer than that in the *trans* isomer.

Based on the experimental data, the rotational energy barrier Δ*G*^‡^ was calculated using Formula (1) [53,54]:

Δ*G^‡^* = *RT_c_* [22.96 + ln(*T_c_*/Δν)] (1)

where *T_c_* represents the coalescence temperature, Δν is chemical shift difference in slow exchange (Hz), and *R* is the gas constant. The rotational energy barriers Δ*G^‡^* were determined to be 10.8 kcal mol^−1^ for *trans*-**Azo-1** and 9.5 kcal mol^−1^ for *cis*-**Azo-1**. This suggests that the rotational impediment of the phthalocyanine rotator diminished when **Azo-1** underwent isomerization from the *trans* configuration to the *cis* configuration.

### 2.5. Density Function Theory Calculations

**Figure 5:**
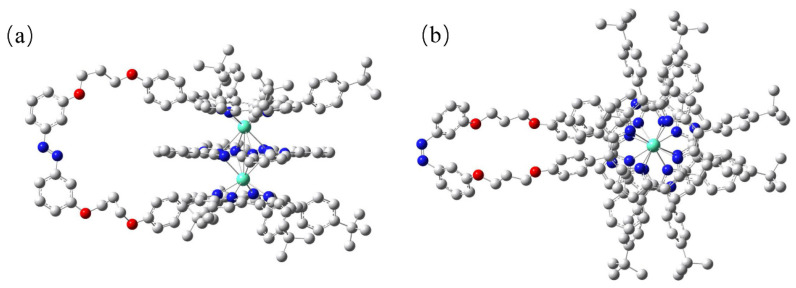
Simulated structures of **Azo-1** (**a**) *trans* configuration and (**b**) *cis* configuration. The grey, red, blue and cyan spherules respectively represent the carbon atom, oxygen atom, nitrogen atom and europium atom.

**Figure 6:**
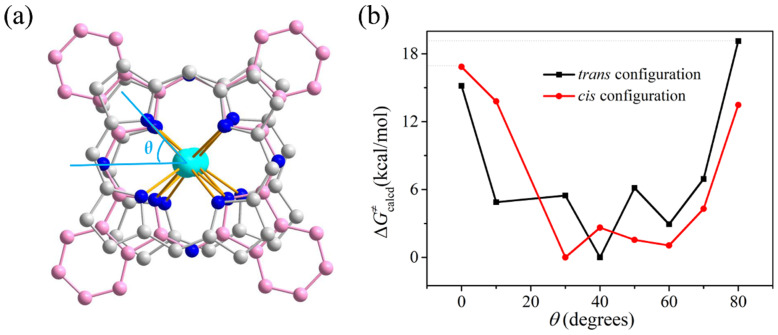
(**a**) Rotational angle *θ* (defined as the projection of the angle formed by aza-nitrogen atom within the phthalocyanine, the central metal atom, and the pyrrole nitrogen atom within the porphyrin). (**b**) Correlation of the calculated rotational barriers Δ*G^‡^_calcd_* with the rotational angle *θ* of **Azo-1** in trans configuration and cis configuration.

Density function theory (DFT) calculations were carried out to further corroborate the experimental results, and the simulated structures of **Azo-1** in *trans* and *cis* configuration are shown in Figure 5. The relationship between the rotational energy barrier and its corresponding rotational angle has been established in Figure 6. The largest rotational energy barrier occurred when the rotational angle was 0° and 80° for *cis* and *trans* configurations, respectively. It was discovered that the rotation pathways of *trans* and *cis* configurations, whereby the benzene ring in phthalocyanine traverses the azobenzene region, possess the greatest rotational energy barrier. The calculated rotational energy barriers were 19.1 and 16.9 kcal mol^−1^ for *trans* and *cis* configurations, respectively. The rotational energy barrier of phthalocyanine rotator in *trans*-**Azo-1** is larger than that in *cis*-**Azo-1**, and this accords with the experimental results. The exhibition of spatial steric hindrance between the azobenzene linkage and the phthalocyanine rotator exerts a profound impact on the rotational dynamics of the rotor. Nevertheless, when azobenzene assumes the *cis* configuration, it is capable of forming a cavity, as depicted in Figure 5, and this is conducive to the passage of phthalocyanine rotors, whence the cis isomer has a lower rotational energy barrier compared to the trans form. Furthermore, the difference between the theoretical and experimental values is likely due to the omission of solvent effect and thermal fluctuation during calculations.

## 3. Experimental Section

### 3.1. Chemicals and Instruments

Column chromatography was carried out on silica gel (200–300 mesh, Qingdao Ocean Chemicals, Qingdao, China) and biobeads (BIORAD S-X1 200–400 mesh, Hercules, CA, USA) with the indicated eluent. Dichloromethane, toluene, and N, N-dimethylformamide (DMF) were freshly distilled from CaH_2_ under nitrogen. n-Octanol was freshly distilled from sodium under nitrogen. 5-(4-hydroxyphenyl)-10,15,20-tris(4-tertbutylphenyl)-porphyrin and Li_2_Pc were synthesized according to the published procedures [55,56]. All other reagents and solvents were used as received.

^1^H NMR spectra were recorded on a Bruker DPX 400 spectrometer (400 MHz) in CDCl_3_ and the chemical shifts were reported relative to internal SiMe_4_. MALDI-TOF mass spectra were recorded using a Bruker (Billerica, MA, USA) MicroflexTM LRF spectrometer with dithranol as the matrix. Electronic absorption spectra were recorded on a Lambda 750 spectrophotometer. *Trans*–*cis*–*trans* isomerization is achieved by means of a UV-LED point light source (IUV-IDS-812) with a spot diameter of 6 mm, an irradiation power of 80%, and an irradiation distance of 2 cm.

All calculations were performed on the basis of PBE0 [57]/BSI level of theory using the Gaussian 09 (revision D.01) software package [58]. The PBE0 functional has been proved suitable for lanthanides [59]. The BSI denotes a mixed basis set, which uses a 6-311G(d) [60] basis set for non-metal elements, and a MWB28 [61] basis set with pseudo potential for europium. Geometry optimizations were performed via the Berny algorithm [62] until the total energy converged to within 1 × 10^−6^ Ha, the forces on all atoms were less than 0.0025 a.u., the maximum step size was less than 0.01 a.u., and the root mean square (RMS) force was less than 0.006 a.u. The relative conformation energy that indicates the coordination stability takes the energy-minimal complex as the reference (0 kJ/mol).

### 3.2. Synthesis and Characterization

#### 3.2.1. Synthesis of 4,4’-Dihydroxylazobenzene (**2**)

To a stirred solution of p-nitrophenol (2.10 g, 15.0 mmol) dissolved in 20 mL of ethanol, a solution of NaOH (2.65 g, 66.2 mmol) dissolved in 5 mL of H_2_O and Zn dust (2.34 g, 36.0 mmol) was subsequently added. The mixture was stirred and heated at reflux for 24 h under nitrogen atmosphere. It was filtered while still hot and the residue was washed with methanol; the evaporation of the filtrate yielded an orange solid. The combined products were then stirred in 2% aq. HCl for 30 min and then filtered. The product was washed 3 times with hot water (15 mL) and the residue was then dried for purification. Then, the residue was purified by column chromatography on silica gel using ethanol/CH_2_Cl_2_ (1:20, in *v*/*v*) as an eluant to yield compound **2** as orange crystals (1.22 g, 76%). ^1^H NMR (DMSO_d6_, 400 MHz, 298 K): *δ* 9.84 (s, 2H), 7.28 (m, 4H), 7.23 (s, 2H), 6.96 (d, 2H).

#### 3.2.2. Synthesis of 4,4’-Di(3-bromopropoxy) Azobenzene (**3**)

A mixture of **2** (536 mg, 2.50 mmol), 1,3-dibromethane (2.55 mL, 25.0 mmol), and anhydrous K_2_CO_3_ (691 mg, 5.00 mmol) in dry DMF (20 mL) was heated to 60 °C and reacted under nitrogen for 24 h. Then, the reaction mixture was poured into water and extracted with CHCl_3_. It was then dried over anhydrous Na_2_SO_4_ and concentrated at reduced pressure. The residue was purified by column chromatography on silica gel with CHCl_3_/n-hexane (3:2, in *v*/*v*) as the eluent. Orange crystals **3** were obtained by recrystallization from CHCl_3_/CH_3_OH (525 mg, 46%). ^1^H NMR (CDCl_3_, 400 MHz, 298 K): *δ* 7.561 (d, 2H), 7.43 (m, 4H), 7.06 (d, 2H), 4.21 (t, 4H), 3.64 (t, 4H), 2.37 (m, 4H).

#### 3.2.3. Synthesis of (TTBPP)Eu(Pc)Eu(TTBPP) (**4**)

TTBPP (160 mg, 0.2 mmol) and Eu(acac)_3_·nH_2_O (150 mg, ca. 0.30 mmol) were refluxed in TCB (6 mL) under a nitrogen atmosphere until the complete formation of europium monoporphyrinate Eu(TTBPP)(acac) (ca. 5 h). The conversion was monitored by UV-Vis spectroscopy. After the complete formation of Eu(TTBPP)(acac), the reaction mixture was cooled to room temperature and Li_2_Pc (104 mg, 0.2 mmol) was added. The mixture was refluxed for a further 8 h. After a brief cooling, the solvent was evaporated under reduced pressure. The residue was purified by column chromatography on silica gel with CHCl_3_ as the eluent. Dark green solid **4** was obtained by recrystallization from CHCl_3_/MeOH (33.8 mg, 14%). ^1^H NMR (CDCl_3_, 400 MHz, 298 K): *δ* 12.70 (s, 8H), 11.38 (s, 8H), 10.61 (s, 8H), 8.97 (s, 2H), 8.46 (s, 6H), 6.69 (s, 6H), 6.17 (s, 2H), 4.74 (d, 8H), 3.96(s, 16H), 1.88 (s, 54H); UV-vis (toluene): λ_max_ (log ε) 353 (5.05), 421 (5.35), 491 (4.63), 555 (4.25), 607 (4.38), 671 (3.90), 955 nm (3.82); MALDI-TOF-MS *m*/*z* calcd. For C_144_H_120_Eu_2_N_16_O_2_: (M^+^) 2410.58; Found: 2410.32.

#### 3.2.4. Synthesis of Azo-(TTBPP)Eu(Pc)Eu(TTBPP) (**Azo-1**)

Anhydrous K_2_CO_3_ (69.0 mg, 0.50 mmol) was added to a flask with dry DMF (10 mL). Then, a mixture solution of **4** (24.10 mg, 0.01 mmol) and **3** (4.56 mg, 0.01 mmol) in dry DMF (10 mL) were added dropwise. The mixture was heated to 90 °C and reacted under nitrogen for 10 h. The mixture was poured into water and extracted with CHCl_3_. It was then dried over anhydrous Na_2_SO_4_ and concentrated at reduced pressure. The product was purified by silica gel column chromatography with dichloromethane/n-hexane (3:2, in *v*/*v*) as the eluent. The first fraction containing the target compound was collected and evaporated. Dark green solid **Azo-1** was obtained by recrystallization from CH_2_Cl_2_/MeOH (5.68 mg, 21%). ^1^H NMR (CDCl_3_, 400 MHz, 298 K): *δ* 12.76 (s, 8H), 11.60 (d, 4H, J = 20.2 Hz), 11.32 (s, 4H), 10.66 (s, 8H), 9.05 (s, 2H), 8.95 (s, 4H), 8.67 (s, 2H), 8.39 (s, 2H), 8.03 (d, 2H, J = 8.4 Hz), 7.84 (t, 2H, J = 8.1 Hz), 7.48 (d, 2H, J = 7.3 Hz), 6.72 (d, 2H, J = 5.8 Hz), 6.66 (d, 4H, J = 5.9 Hz), 6.43 (d, 2H, J = 5.5 Hz), 5.07 (d, 2H, J = 20.1 Hz), 5.01 (s, 4H), 4.91 (s, 4H), 4.80 (d, 2H, J = 6.6 Hz), 4.67 (d, 4H, J = 6.2 Hz), 4.02 (d, 8H, J = 16.2 Hz), 3.91 (d, 8H, J = 16.4 Hz), 2.85 (s, 4H), 1.92 (s, 54H); UV-vis (toluene): λ_max_ (log *ε*): 354 (5.05), 421 (5.33), 492 (4.59), 555 (4.16), 607 (4.30), 957 nm (3.77); MALDI-TOF-MS *m*/*z* calcd. for C_162_H_138_Eu_2_N_18_O_4_ (MH^+^) 2704.94, found 2706.08.

## 4. Conclusions

A molecular rotor based on a mixed (phthalocyaninato)(porphyrinato) rare earth triple-decker complex, with the azobenzene-linked bisporphyrin as the stator and the phthalocyanine as the rotator was reported. The reversible *cis*/*trans* isomerization of the azobenzene linker in the rotor was realized by alternative irradiation with ultraviolet and visible light. The rotational behaviors of the phthalocyanine rotator in *cis* and *trans* isomers were investigated by VT-^1^H NMR analysis, indicating that the rotor possesses a smaller rotational energy barrier in the *cis* configuration than that in the *trans* configuration, which has also been supported by DFT calculations. This work demonstrates a new strategy for the modulation of the intramolecular rotational energy barrier by reversible photo-isomerization and thus can be helpful for the design of photocontrollable molecular rotors.

## Data Availability

The data supporting this article have been included as part of the Supplementary Materials.

## Supplementary Materials

The following supporting information can be downloaded at: https://www.mdpi.com/article/10.3390/molecules30020326/s1, Figure S1: Experimental mass spectrum for the protonated molecular ion of **Azo-1**; Figure S2: ^1^H NMR spectrum of **4** in CDCl_3_ at 298 K. Figure S3: ^1^H–^1^H COSY spectra of **4** in CDCl_3_ at 298 K; Figure S4: ^1^H NMR spectrum of **Azo-1** in CDCl_3_ at 298 K; Figure S5: ^1^H–^1^H COSY spectra of **Azo-1** in CDCl_3_ at 298 K; Figure S6: Electron absorption spectra of compound **4** at 298 K; Figure S7: Electronic absorption spectra (a) and absorption changes at λ = 451 nm (b) of **Azo-1** under light irradiation at λ = 365 nm or λ = 450 nm; Figure S8: Electronic absorption spectra of (TTBPP)Eu(Pc)Eu(TTBPP) (compound **4**), under light irradiation at λ = 365 nm for 10 and 20 min respectively; Figure S9: ^1^H NMR spectra of **Azo-1** in trans configuration in the range of 205–298 K; Figure S10: ^1^H NMR spectra of **Azo-1** in *cis* configuration in the range of 205–298 K.

## References

[1] Goodsell D.S. (2009). The Machinery of Life.

[2] Erbas-Cakmak S., Leigh D.A., McTernan C.T., Nussbaumer A.L. (2015). Artificial Molecular Machines. Chem. Rev..

[3] Dattler D., Fuks G., Heiser J., Moulin E., Perrot A., Yao X.Y., Giuseppone N. (2020). Design of Collective Motions from Synthetic Molecular Switches, Rotors, and Motors. Chem. Rev..

[4] Chen S., Wang Y., Nie T., Bao C., Wang C., Xu T., Lin Q., Qu D.-H., Gong X., Yang Y. (2018). An Artificial Molecular Shuttle Operates in Lipid Bilayers for Ion Transport. J. Am. Chem. Soc..

[5] Krause S., Feringa B.L. (2020). Towards artificial molecular factories from framework-embedded molecular machines. Nat. Rev. Chem..

[6] Goswami A., Saha S., Elramadi E., Ghosh A., Schmittel M. (2021). Off-Equilibrium Speed Control of a Multistage Molecular Rotor: 2-Fold Chemical Fueling by Acid or Silver(I). J. Am. Chem. Soc..

[7] Wu Y., Wang G., Li Q., Xiang J., Jiang H., Wang Y. (2018). A multistage rotational speed changing molecular rotor regulated by pH and metal cations. Nat. Commun..

[8] Civita D., Kolmer M., Simpson G.J., Li A.P., Hecht S., Grill L. (2020). Control of long-distance motion of single molecules on a surface. Science.

[9] Zhou Q.H., Chen J.W., Luan Y.F., Vainikka P.A., Thallmair S., Marrink S.J., Feringa B.L., Rijn P.V. (2020). Unidirectional rotating molecular motors dynamically interact with adsorbed proteins to direct the fate of mesenchymal stem cells. Sci. Adv..

[10] Dong J.Q., Wee V., Peh S.B., Zhao D. (2021). Molecular-Rotor-Driven Advanced Porous Materials. Angew. Chem. Int. Ed..

[11] Bracco S., Miyano T., Negroni M., Bassanetti I., Marchio L., Sozzani P., Tohnai N., Comotti A. (2017). CO_2_ regulates molecular rotor dynamics in porous materials. Chem. Commun..

[12] Schmitt S., Renzer G., Benrath J., Best A., Jiang S., Landfester K., Butt H.J., Simonutti R., Crespy D., Koynov K. (2022). Monitoring the Formation of Polymer Nanoparticles with Fluorescent Molecular Rotors. Macromolecules.

[13] Ellis E., Moorthy S., Chio W.I.K., Lee T.C. (2018). Artificial molecular and nanostructures for advanced nanomachinery. Chem. Commun..

[14] Kaleta J., Kaletová E., Císařová I., Teat S.J., Michl J. (2015). Synthesis of Triptycene-Based Molecular Rotors for Langmuir Blodgett Monolayers. J. Org. Chem..

[15] Páez-Pérez M., López-Duarte I., Vyšniauskas A., Brooks N.J., Kuimova M.K. (2021). Imaging non-classical mechanical responses of lipid membranes using molecular rotors. Chem. Sci..

[16] Ma L.J., Geng Y.J., Zhang G.Y., Hu Z.W., James T.D., Wang X.F., Wang Z. (2023). Near-Infrared Bodipy-Based Molecular Rotors for *β*-Amyloid Imaging In Vivo. Adv. Healthc. Mater..

[17] Fares M., Li Y., Liu Y., Miao K., Gao Z., Zhai Y., Zhang X. (2018). A Molecular Rotor-Based Halo-Tag Ligand Enables a Fluorogenic Proteome Stress Sensor to Detect Protein Misfolding in Mildly Stressed Proteome. Bioconjug. Chem..

[18] Chen W., Han J., She J., Wang F., Zhu L., Yu R.-Q., Jiang J.H. (2020). Simultaneous imaging of lysosomal and mitochondrial viscosity during mitophagy using molecular rotors with dual-color emission. Chem. Commun..

[19] Prando G., Perego J., Negroni M., Riccò M., Bracco S., Comotti A., Sozzani P., Carretta P. (2020). Molecular Rotors in a Metal-Organic Framework: Muons on a Hyper-Fast Carousel. Nano Lett..

[20] Perego J., Bezuidenhout C.X., Bracco S., Piva S., Prando G., Aloisi C., Carretta P., Kaleta J., Le T.P., Sozzani P. (2023). Benchmark Dynamics of Dipolar Molecular Rotors in Fluorinated Metal-Organic Frameworks. Angew. Chem. Int. Ed..

[21] Li J., Wan Y., Jiang G., Ozaki Y., Pi F. (2024). Hygrosensitive Dynamic Covalent Organic Framework Films: Harnessing Molecular Rotors for Moisture-Driven Actuation in Wearable Health Monitoring. Adv. Funct. Mater..

[22] Tsurunaga M., Inagaki Y., Momma H., Kwon E., Yamaguchi K., Yoza K., Setaka W. (2018). Dielectric Relaxation of Powdered Molecular Gyrotops Having a Thiophene Dioxide-diyl as a Dipolar Rotor. Org. Lett..

[23] Tsuchiya T., Inagaki Y., Yamaguchi K., Setaka W. (2021). Structure and Dynamics of Crystalline Molecular Gyrotops with a Difluorophenylene Rotor. J. Org. Chem..

[24] Yu C., Ma L., He J., Xiang J., Deng X., Wang Y., Chen X., Jiang H. (2016). Flexible, Linear Chains Act as Baffles to Inhibit the Intramolecular Rotation of Molecular Turnstiles. J. Am. Chem. Soc..

[25] Dong J.Q., Li X., Zhang K., Yuan Y.D., Wang Y.X., Zhai L.Z., Liu G.L., Yuan D.Q., Jiang J.W., Zhao D. (2018). Confinement of Aggregation-Induced Emission Molecular Rotors in Ultrathin Two-Dimensional Porous Organic Nanosheets for Enhanced Molecular Recognition. J. Am. Chem. Soc..

[26] Dong J.Q., Zhang K., Li X., Qian Y.H., Zhu H., Yuan D.Q., Xu Q.H., Jiang J.W., Zhao D. (2017). Ultrathin two-dimensional porous organic nanosheets with molecular rotors for chemical sensing. Nat. Commun..

[27] Paez-Perez M., Kuimova M.K. (2023). Molecular Rotors: Fluorescent Sensors for Microviscosity and Conformation of Biomolecules. Angew. Chem. Int. Ed..

[28] Polita A., Toliautas S., Žvirblis R., Vyšniauskas A. (2020). The effect of solvent polarity and macromolecular crowding on the viscosity sensitivity of a molecular rotor BODIPY-C10. Phys. Chem. Chem. Phys..

[29] Bai Y., Liu Y. (2021). Illuminating Protein Phase Separation: Reviewing Aggregation-Induced Emission, Fluorescent Molecular Rotor and Solvatochromic Fluorophore Based Probes. Chem. Eur. J..

[30] Wu Y.L., Frasconi M., Liu W.G., Young R.M., Goddard W.A., Wasielewski M.R., Stoddart J.F. (2020). Electrochemical Switching of a Fluorescent Molecular Rotor Embedded within a Bistable Rotaxane. J. Am. Chem. Soc..

[31] Inukai M., Tamura M., Horike S., Higuchi M., Kitagawa S., Nakamura K. (2018). Storage of CO into Porous Coordination Polymer Controlled by Molecular Rotor Dynamics. Angew. Chem. Int. Ed..

[32] Feng L., Xie Y., Au-Yeung S.K., Hailu H.B., Liu Z., Chen Q., Zhang J., Pang Q., Yao X., Yang M. (2020). A fluorescent molecular rotor probe for tracking plasma membranes and exosomes in living cells. Chem. Commun..

[33] Fam K.T., Saladin L., Klymchenko A.S., Collot M. (2021). Confronting molecular rotors and self-quenched dimers as fluorogenic BODIPY systems to probe biotin receptors in cancer cells. Chem. Commun..

[34] Zsila F. (2021). Comment on “Emissive H-Aggregates of an Ultrafast Molecular Rotor: A Promising Platform for Sensing Heparin”. ACS Appl. Mater. Inter..

[35] Szymański W., Beierle J.M., Kistemaker H.A.V., Velema W.A., Feringa B.L. (2013). Reversible Photocontrol of Biological Systems by the Incorporation of Molecular Photoswitches. Chem. Rev..

[36] Tylkowski B., Trojanowska A., Marturano V., Nowak M., Marciniak L., Giamberini M., Ambrogi V., Cerruti P. (2017). Power of light-Functional complexes based on azobenzene molecules. Coord. Chem. Rev..

[37] Adam A., Haberhauer G. (2017). Switching Process Consisting of Three Isomeric States of an Azobenzene Unit. J. Am. Chem. Soc..

[38] Islam Sk A., Kundu K., Kundu P.K. (2020). Azobenzene Isomerization-Induced Photomodulation of Electronic Properties of N-Heterocyclic Carbenes. Chem. Eur. J..

[39] Kawamoto M., Shiga N., Takaishi K., Yamashita T. (2010). Non-destructive erasable molecular switches and memory using light-driven twisting motions. Chem. Commun..

[40] Bandara H.M.D., Friss T.R., Enriquez M.M., Isley W., Incarvito C., Frank H.A., Gascon J., Burdette S.C. (2010). Proof for the Concerted Inversion Mechanism in the trans→cis Isomerization of Azobenzene Using Hydrogen Bonding to Induce Isomer Locking. J. Org. Chem..

[41] Tkachenko I.M., Kurioz Y.I., Kravchuk R.M., Kobzar Y.L., Litoshenko D.V., Glushchenko A.V., Shevchenko V.V., Nazarenko V.G. (2024). Photoinduced Birefringence and Liquid Crystal Orientation on Polymers with Different Azobenzene Content in the Main Chain. ACS Appl. Mater. Inter..

[42] Zheng M., Yuan J. (2022). Polymeric nanostructures based on azobenzene and their biomedical applications: Synthesis, self-assembly and stimuli-responsiveness. Org. Biomol. Chem..

[43] Cheng H.B., Zhang S., Qi J., Liang X.J., Yoon J. (2021). Advances in Application of Azobenzene as a Trigger in Biomedicine: Molecular Design and Spontaneous Assembly. Adv. Mater..

[44] Zhang L., Liu H., Du Q., Zhang G., Zhu S., Wu Z., Luo X. (2022). Photoliquefiable Azobenzene Surfactants toward Solar Thermal Fuels that Upgrade Photon Energy Storage via Molecular Design. Small.

[45] Liu C., Yang W., Zhang Y.X., Jiang J.Z. (2020). Quintuple-Decker Heteroleptic Phthalocyanine Heterometallic Samarium-Cadmium Complexes. Synthesis, Crystal Structure, Electrochemical Behavior, and Spectroscopic Investigation. Inorg. Chem..

[46] Liu W.B., Pan H.H., Wang Z.Q., Wang K., Qi D.D., Jiang J.Z. (2017). Sandwich rare earth complexes simultaneously involving aromatic phthalocyanine and antiaromatic hemiporphyrazine ligands showing a predominantly aromatic nature. Chem. Commun..

[47] Martynov A.G., Horii Y., Katoh K., Bian Y.Z., Jiang J.Z., Yamashita M., Gorbunova Y.G. (2022). Rare-earth based tetrapyrrolic sandwiches: Chemistry, materials and applications. Chem. Soc. Rev..

[48] Tashiro K., Konishi K., Aida T. (1997). Enantiomeric Resolution of Chiral Metallobis(porphyrin)s: Studies on Rotatability of Electronically Coupled Porphyrin Ligands. Angew. Chem. Int. Ed..

[49] Tashiro K., Fujiwara T., Konishi K., Aida T. (1998). Rotational oscillation of two interlocked porphyrins in cerium bis(5,15-diarylporphyrinate) double-deckers. Chem. Commun..

[50] Bian Y., Zhang Y., Ou Z., Jiang J., Kadish K.M., Smith K.M., Guilard R. (2011). Handbook of Porphyrin Science.

[51] Sun X., Li R., Wang D., Dou J., Zhu P., Lu F., Ma C., Choi C.F., Cheng D.Y.Y., Ng D.K.P. (2004). Synthesis and Characterization of Mixed Phthalocyaninato and meso-Tetrakis (4-chlorophenyl) porphyrinato Triple-Decker Complexes-Revealing the Origin of Their Electronic Absorptions. Eur. J. Inorg. Chem..

[52] Das G., Prakasam T., Addicoat M.A., Sharma S.K., Rayaux F., Mathew R., Baias M., Jagannathan R., Olson M.A., Trabolsi A. (2019). Azobenzene-Equipped Covalent Organic Framework: Light-Operated Reservoir. J. Am. Chem. Soc..

[53] Birin K.P., Gorbunova Y.G., Tsivadze A.Y. (2011). NMR investigation of intramolecular dynamics of heteroleptic triple-decker (porphyrinato)(phthalocyaninato) lanthanides. Dalton Trans..

[54] Huggins M.T., Kesharwani T., Buttrick J., Nicholson C. (2020). Variable Temperature NMR Experiment Studying Restricted Bond Rotation. J. Chem. Educ..

[55] Lu W.X., Li L.K., Yang H.F., Zhao L.Y., Qi D.D., Bian Y.Z., Jiang J.Z. (2017). Intramolecular chirality induction and intermolecular chirality modulation in BINOL bridged bisporphyrin hosts. Dyes Pigments.

[56] Cao W., Wang H., Wang X., Lee H.K., Ng D.K.P., Jiang J. (2012). Constructing Sandwich-Type Rare Earth Double-Decker Complexes with N-Confused Porphyrinato and Phthalocyaninato Ligands. Inorg. Chem..

[57] Adamo C., Barone V. (1999). Toward reliable density functional methods without adjustable parameters: The PBE0 mode. J. Chem. Phys..

[58] Frisch M.J., Trucks G.W., Schlegel H.B., Scuseria G.E., Robb M.A., Cheeseman J.R., Scalmani G., Barone V., Mennucci B., Petersson G.A. (2013). Gaussian 09, Revision E.01.

[59] Chen X., Chen T.T., Li W.L., Lu J.B., Zhao L.J., Jian T., Hu H.S., Wang L.S., Li J. (2019). Lanthanides with Unusually Low Oxidation States in the PrB_3_– and PrB_4_– Boride Clusters. Inorg. Chem..

[60] McLean A.D., Chandler G.S. (1980). Contracted Gaussian basis sets for molecular calculations. I. Second row atoms, Z=11–18. J. Chem. Phys..

[61] Dunning T.H., Hay P.J., Schaefer H.F. (1977). Modern Theoretical Chemistry.

[62] Peng C., Ayala P.Y., Schlegel H.B., Frisch M.J. (1996). Using redundant internal coordinates to optimize equilibrium geometries and transition states. J. Comput. Chem..

