# Supplementary material for: A Photocontrolled Molecular Rotor Based on Azobenzene-Strapped Mixed (Phthalocyaninato)(Porphyrinato) Rare Earth Triple-Decker
Source: Molecules. 2025 Jan 15;30(2):326. doi: 10.3390/molecules30020326 (PMC11767808; doi:10.3390/molecules30020326)
Supplement: Supplementary file 1 [file molecules-30-00326-s001.zip › molecules-3392541-supplementary.pdf]

Supporting Information

A Photocontrolled Molecular Rotor based on Azobenzene-strapped Mixed (Phthalocyaninato)(Porphyrinato) Rare Earth Tripledecker

Wenxin Lu[a,b], Tiantian Mu[a], Yuehong Zhang[c], Bo Chen[a], Huantao Guo[a], Luyang Zhao[d], Peng Wang,[a,*] and Yongzhong Bian[b,*]

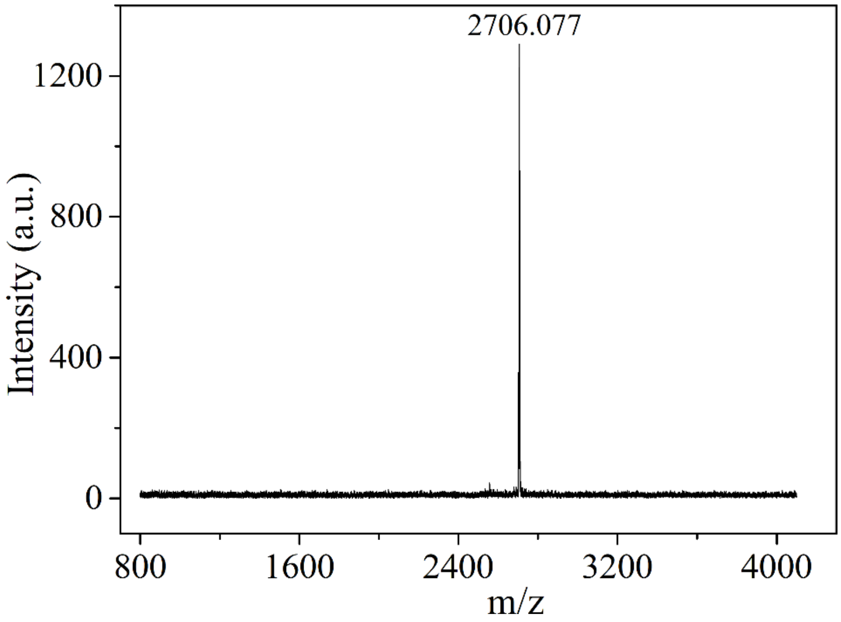

Figure S1. Experimental mass spectrum for the protonated molecular ion of **Azo-1**.

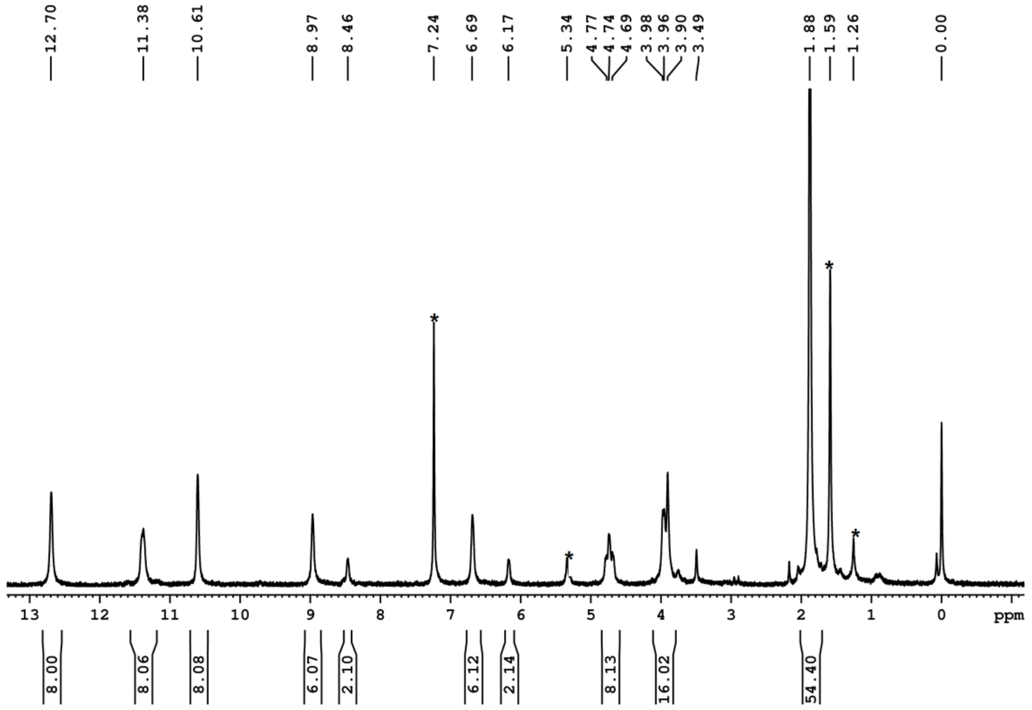

Figure S2. ^{1}H NMR spectrum of **4** in $CDCl_3$ at 298 K. * indicate the residual solvent and H_2O signals.

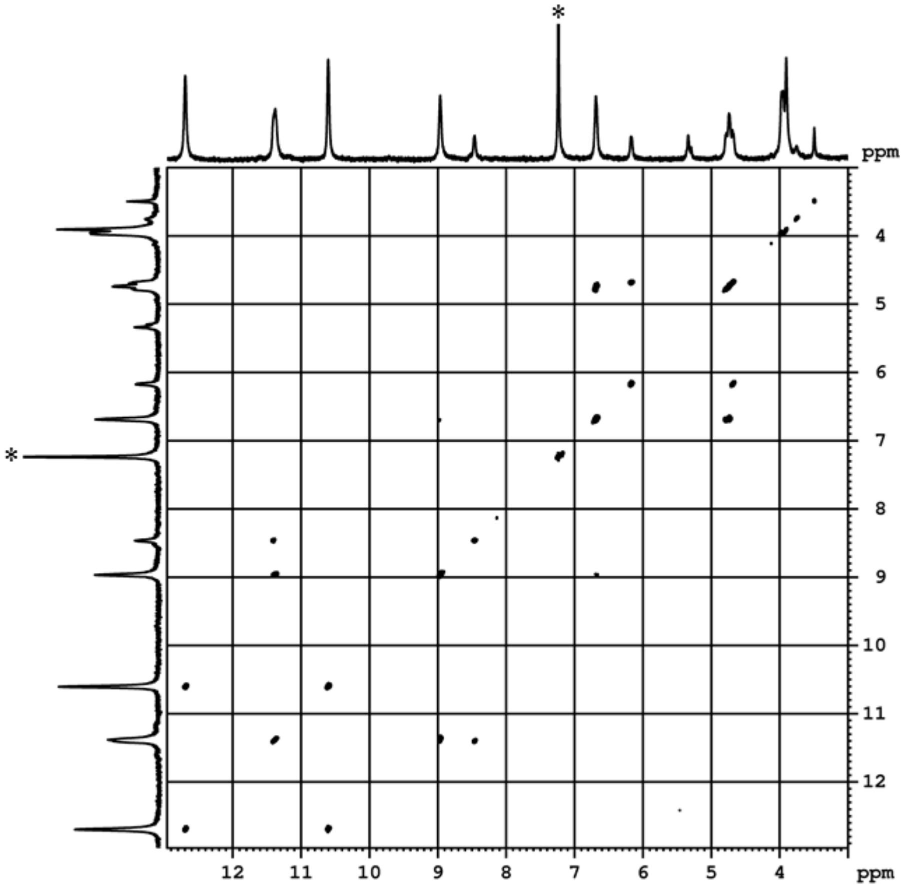

Figure S3. ^{1}H-^{1}H COSY spectra of **4** in $CDCl_3$ at 298 K. * indicate the residual solvent signals.

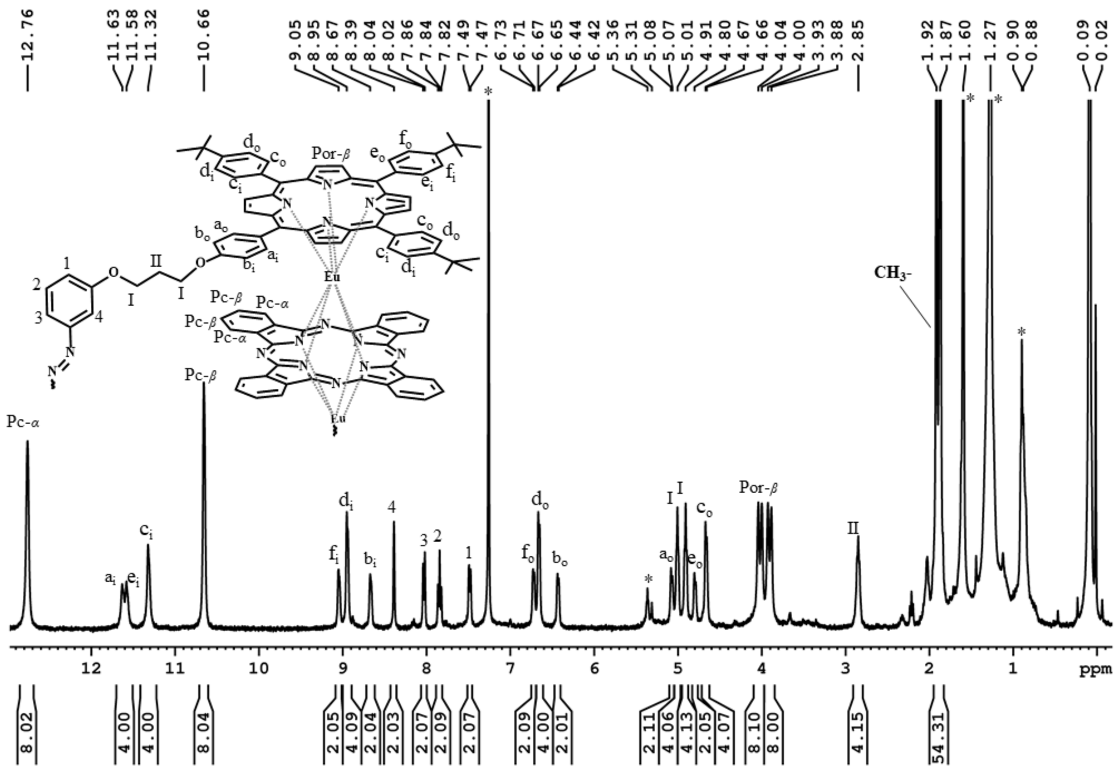

Figure S4. ^{1}H NMR spectrum of **Azo-1** in $CDCl_3$ at 298 K. * indicate the residual solvent and H_2O signals.

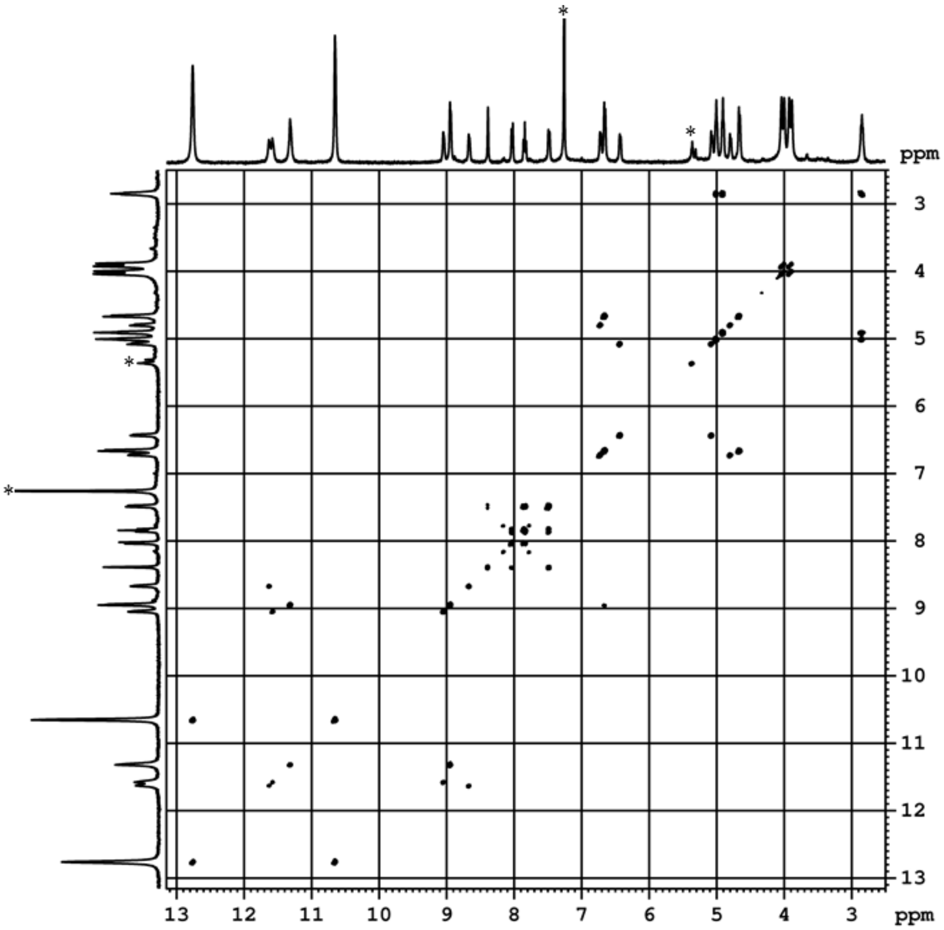

Figure S5. ^{1}H-^{1}H COSY spectra of **Azo-1** in $CDCl_3$ at 298 K. * indicate the residual solvent signals.

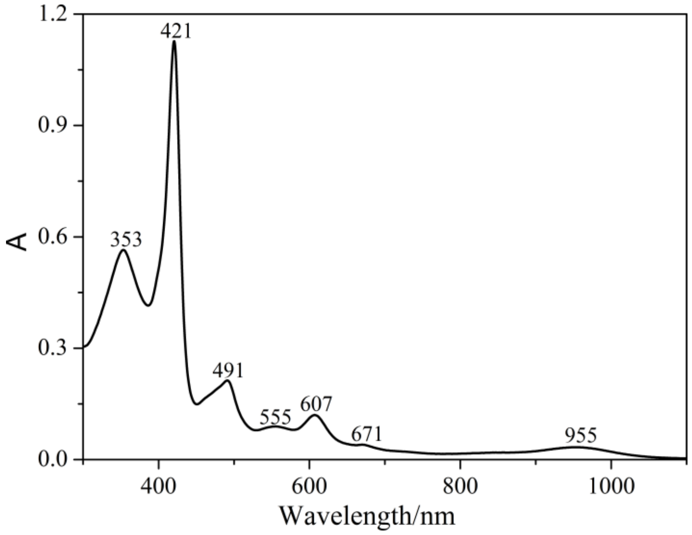

Figure S6. Electron absorption spectra of compound **4** at 298 K (5×10^{-6} M in toluene).

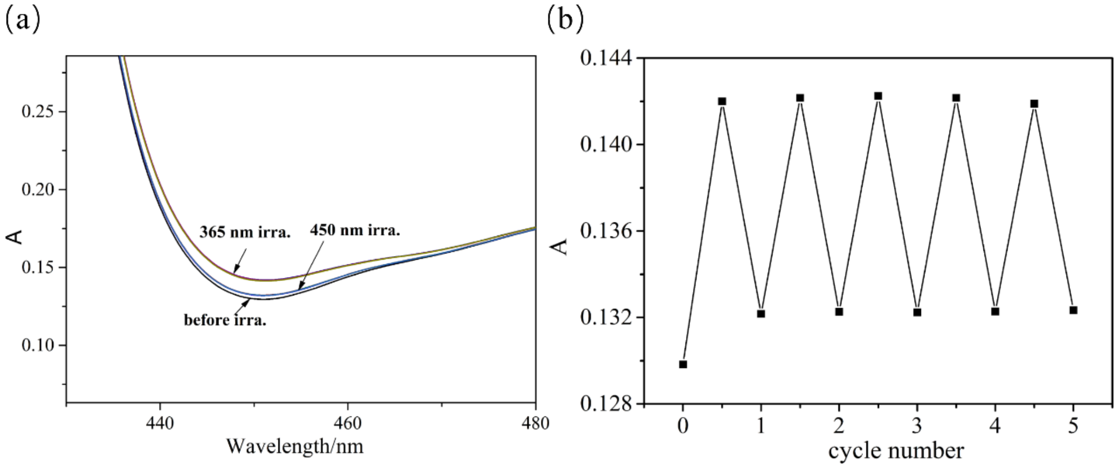

Figure S7. Electronic absorption spectra (a) and absorption changes at λ = 451 nm (b) of **Azo-1** (5.0×10^{-6} M in $CHCl_3$) under light irradiation at λ = 365 nm or λ = 450 nm.

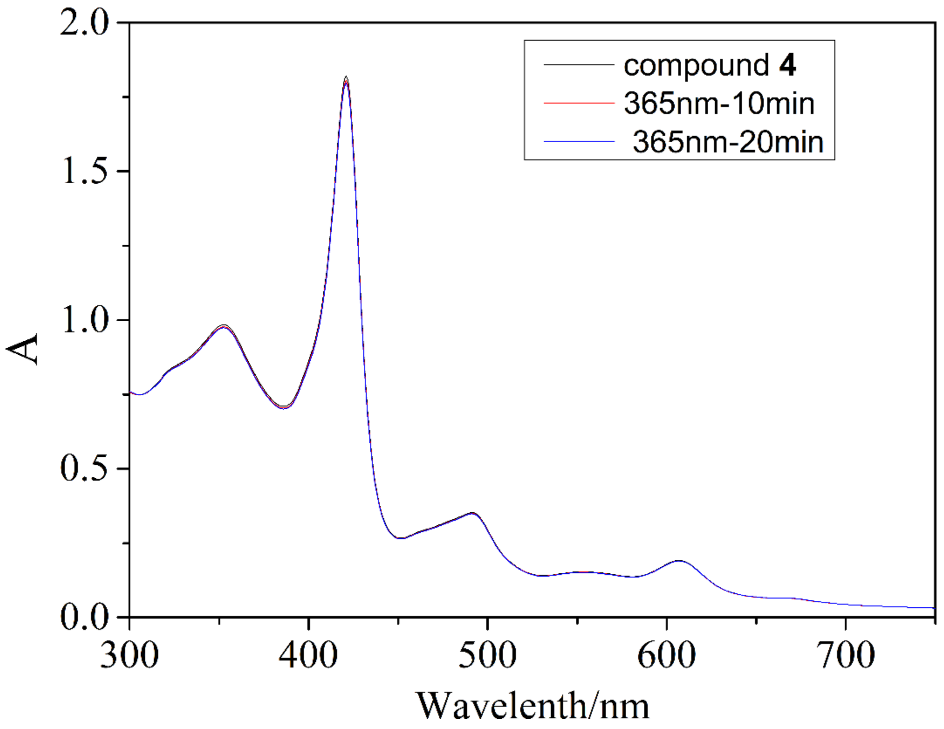

Figure S8. Electronic absorption spectra of (TTBPP)Eu(Pc)Eu(TTBPP) (compound **4**)(in toluene), under light irradiation at λ = 365 nm for 10 and 20 minutes respectively.

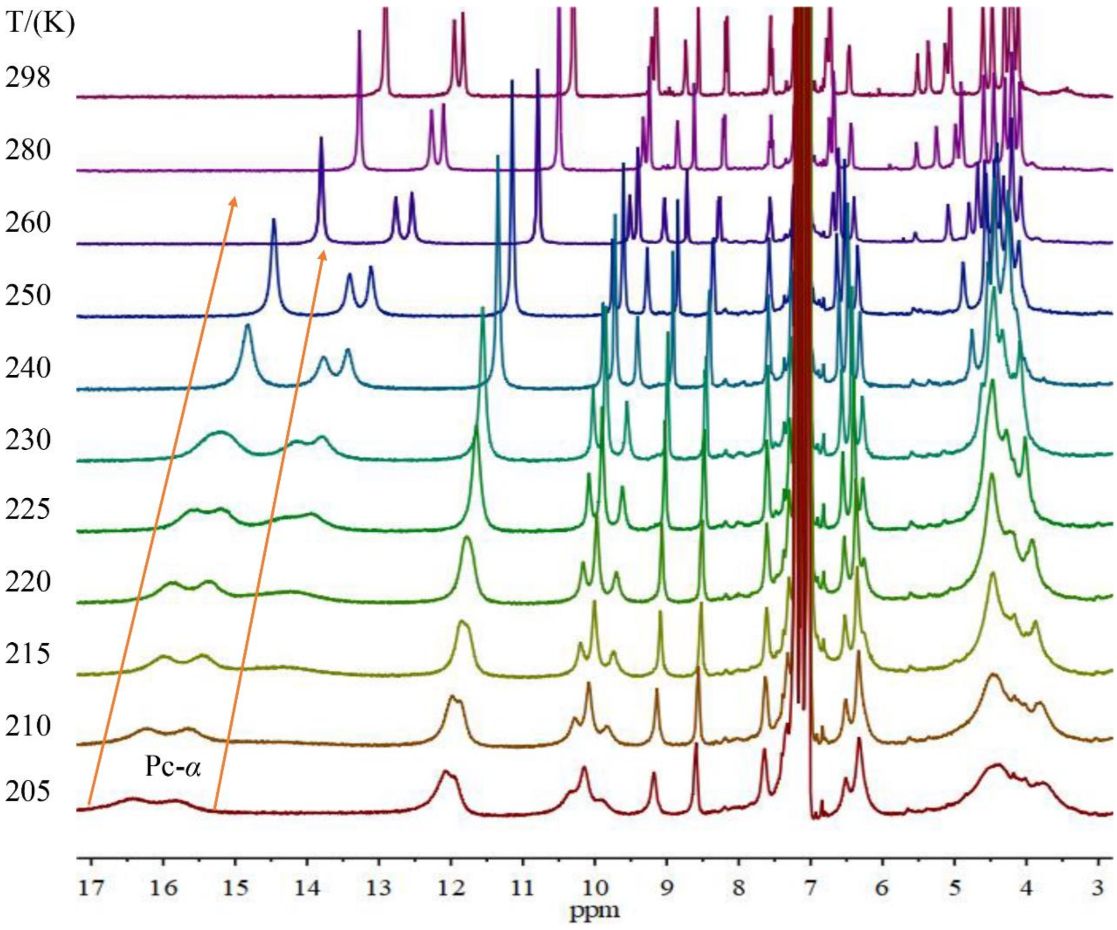

Figure S9. ^{1}H NMR spectra of **Azo-1** in *trans* configuration ([D8] toluene) in the range of 205 K-298 K.

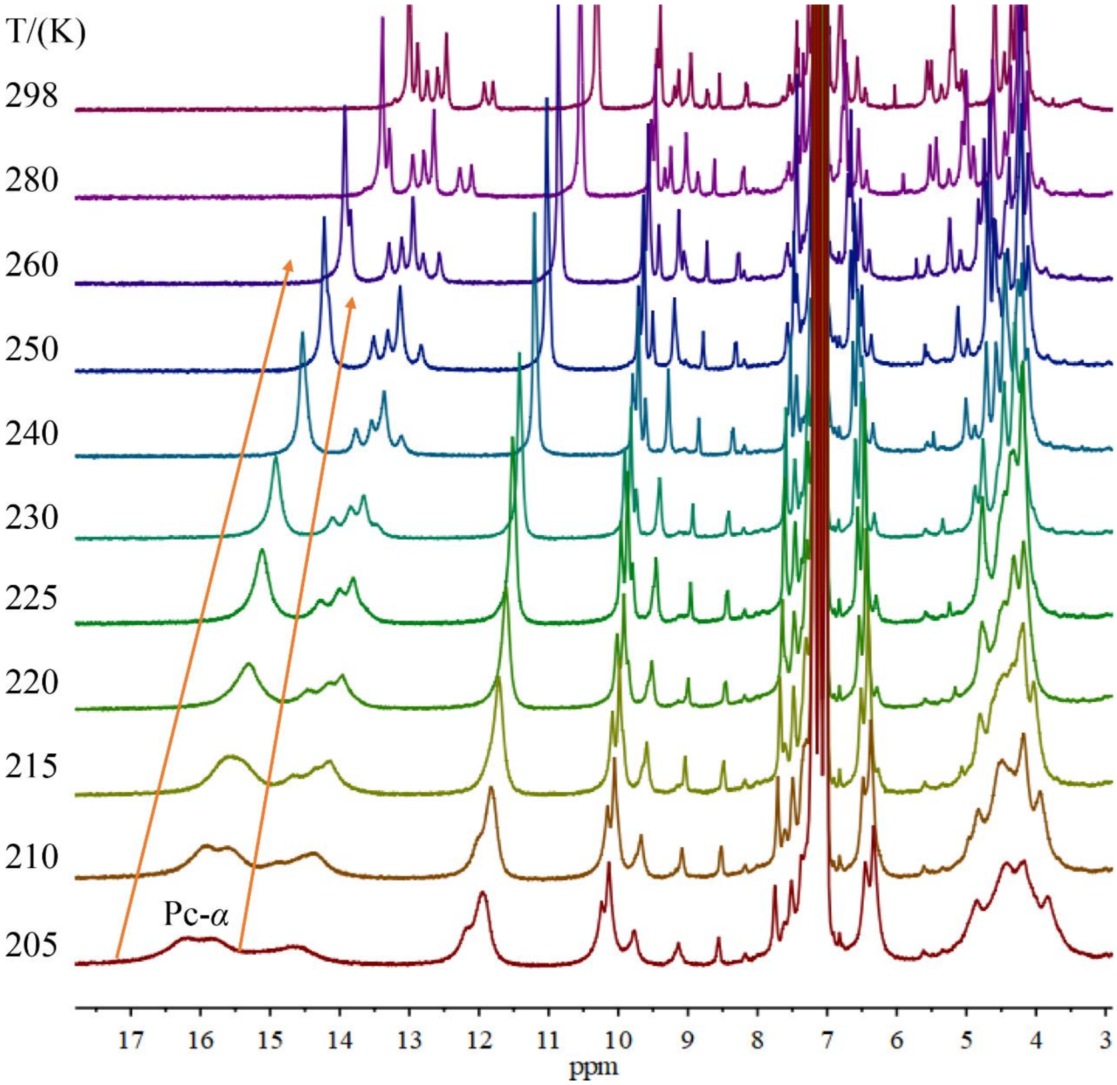

Figure S10. ^{1}H NMR spectra of **Azo-1** in *cis* configuration ([D8] toluene) in the range of 205 K-298 K.
